# Effect of Probiotics on Allergic Rhinitis: A Randomized, Controlled, Clinical Trial

**DOI:** 10.31661/gmj.v9i0.1918

**Published:** 2020-06-26

**Authors:** Mahnaz Sadeghi-Shabestari, Yalda Jabbari Moghaddam, Hasan Rezapoor, Mojataba Sohrabpour

**Affiliations:** ^1^Immunology Research Center of Tabriz, TB and lung Research Center of Tabriz, Tabriz University of Medical Sciences, Tabriz, Iran; ^2^ENT Department, Tabriz University of Medical Sciences, Tabriz, Iran; ^3^Noncommunicable Diseases Research Center, Fasa University of Medical Sciences, Fasa, Iran

**Keywords:** Probiotics, Allergic Rhinitis, Adult

## Abstract

**Background:**

Allergic rhinitis (AR) is one of the most common diseases in the world and affects about 10-50% of the general population. Probiotics are live microorganisms that help the normal state of the intestine, and if prescribed correctly, they can stimulate the mucosal immune system to prevent inflammatory symptoms of allergy and atopy. The present study aims to investigate the role of probiotics in the treatment of AR when added to standard therapy as adjuvant agents.

**Materials and Methods:**

In this clinical trial study, 28 patients older than 15 years with AR randomly divided into probiotics and control groups. The probiotics group received standard therapy for AR accompanied by probiotic capsules every 12 hours for eight weeks, whereas the control group received standard therapy for AR with placebo capsules as the same protocol. Data were analyzed using SPSS Version 23 (IBM Corporation, Armonk, NY, USA) and, the P-value less than 0.05 was considered statistically significant.

**Results:**

In the probiotics group, 14.3% of patients had sneezing at the baseline, which significantly decreased to 4.6% (P<0.01). Also, the necessity for nasal and oral corticosteroids after treatment with probiotics in the probiotics group was less than the control group (P<0.01). Although cough, nasal discharge, conchae hypertrophy, and night sleep disorders reduced after treatment with probiotics, this reduction was not statistically significant between the two groups.

**Conclusion:**

Based on the results of this clinical trial, the use of probiotics had no significant effect on the outcome of patients with AR.

## Introduction

Allergic rhinitis (AR) is one of the most common diseases in the world and affects about 10-50% of the genaceral population. According to recent evidence, the estimated prevalence of AR and asthma is 20% to 30%. It affects the patient’s health and negatively affects the quality of sleep and function of the individual and many annual expenditures for controlling the diseases that are consumed by the individual (1). Histamine is the most common mediator of AR, and antihistamines are prescribed as a first-priority drug for the control of the disease. AR by symptoms such as sneezing, itching, sleep disturbances, insomnia, learning difficulties, increased drowsiness, and reduced productivity and concentration on work and school activities has effects even more than common illnesses such as depression, migraine, respiratory infections, diabetes, and cardiovascular disease. AR is an example of type I hypersensitivity (IgE-mediated disease) that acts by degranulation of mast cells (2). The agent responsible for the reaction is the mast cells’ abandoned mediators and basophils. Annually the cost for control of AR is more than chronic and prevalent diseases such as diabetes and upper respiratory tract infections (3). Probiotics are live microorganisms that help the normal state of the intestine, and they can stimulate the mucosal immune system to prevent inflammatory symptoms of allergy and atopy (4). The efficacy of probiotics in comparison with conventional treatments, such as old and new generations of antihistamines, anti-leukotriene, topical and systemic corticosteroids, and immunotherapy are not specified (5). Although probiotics stimulate the immune system at the surface of the mucosa and prevent the onset of atopy and inflammatory reactions caused by AR disease, there is no consensus even with the anti-inflammatory effects [4]. The present study aims to investigate the role of probiotics in combination with standard therapy while using one of the therapeutic agents for AR. On the other hand, by control of disease, no cost will be imposed on the patient and family

## Materials and Methods

 In this randomized, controlled clinical trial study, we included new cases of AR patients older than 15 years that have referred to Allergy and ENT clinics Tabriz University of Medical Sciences.

###  Sample Size

 The sample size estimated by using software calculations with the admission of 5% error and 80% to 90% of power was 60 patients who were divided into two groups as probiotics and control. Patients with a history of corticosteroid intake in the last two weeks or antibiotics in the last four weeks before entering the study, history of food allergy, lactose intolerance, the prohibition of probiotic use, chronic diseases, other types of rhinitis and respiratory diseases were excluded of study.

###  Randomization and Study Groups

After diagnosis and selection of AR patients, they were randomly divided into two probiotics or intervention and control groups according to Block Randomization as quadruple blocks and also by the randomized blind tables. One group, in addition to receiving a routine diet therapy, also received a probiotics capsule every 12 hours for eight weeks, and the control group received a placebo in the same protocol in addition to routine treatment. Probiotic capsule FamiLact supplement (Zist takhmir company, Tehran, Iran) as a probiotic supplement was used in this study. The shape of the placebo was the same as real probiotics, which provided by the company. The strains used in this supplement were Kant’s 109 CFU probiotics, including strains of *Lactobacillus Casei*, *L. acidophilus*, *L. Rhamnosus*, *L. Bulgaricus*, *Bifidobacterium Breve*, *B. Longum*, *Streptococcus Thermophilus*, and Fructooligosaccharides. Patients demographic information including age, sex, place of residence, type of AR (persistent or intermittent), occupational exposure to contaminants, having other diseases (including asthma, eczema, hives, smoking, coughing, sneezing, throat and nose scrub, night snoring, open mouth breathing) and also clinical symptoms of patients (including nasal polyps, lower conchae hypertrophy, rhinorrhea, posterior secretion, chest alterations, and Waters view findings) were examined and recorded by a specialist in allergy and clinical immunology and the skin prick test was also performed. All patients were examined and followed-up every two months during the study, and data were collected through a checklist of patients’ files and also by a face-to-face interview. On the other hand, all examination and follow-ups of patients were performed by the same subspecialist.

###  Statical Analysis

 To describe the descriptive variables, descriptive analysis tests, including mean, standard deviation (SD), and frequency percentages were used. All patients examined before and after drug usage, and all findings collected. A Chi-square test was used to compare the qualitative variables. Data were analyzed using SPSS Ver. 23 statistical analyses were performed. A P-value less than 0.05 was considered statistically significant.

###  Research Ethics, Consent, and Permissions

 This study was approved by the Ethics Committee of Tabriz University of Medical Sciences (approval code: IR.TBZMED.REC.1396.461) and registered by the Clinical Trials Registry (IRCT code: IRCT20161215031429N3).

## Results

**Figure 1:**
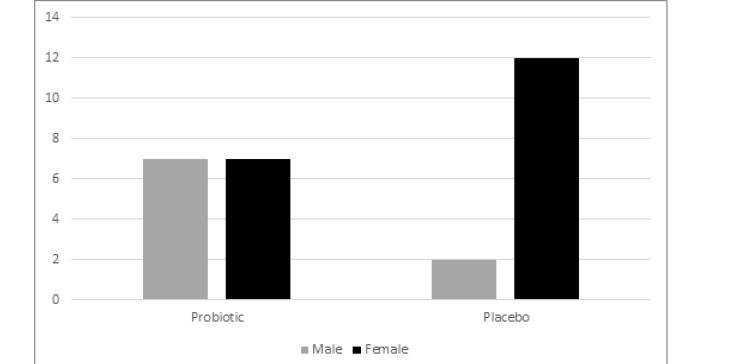
Comparison of gender between two Groups

**Figure 2:**
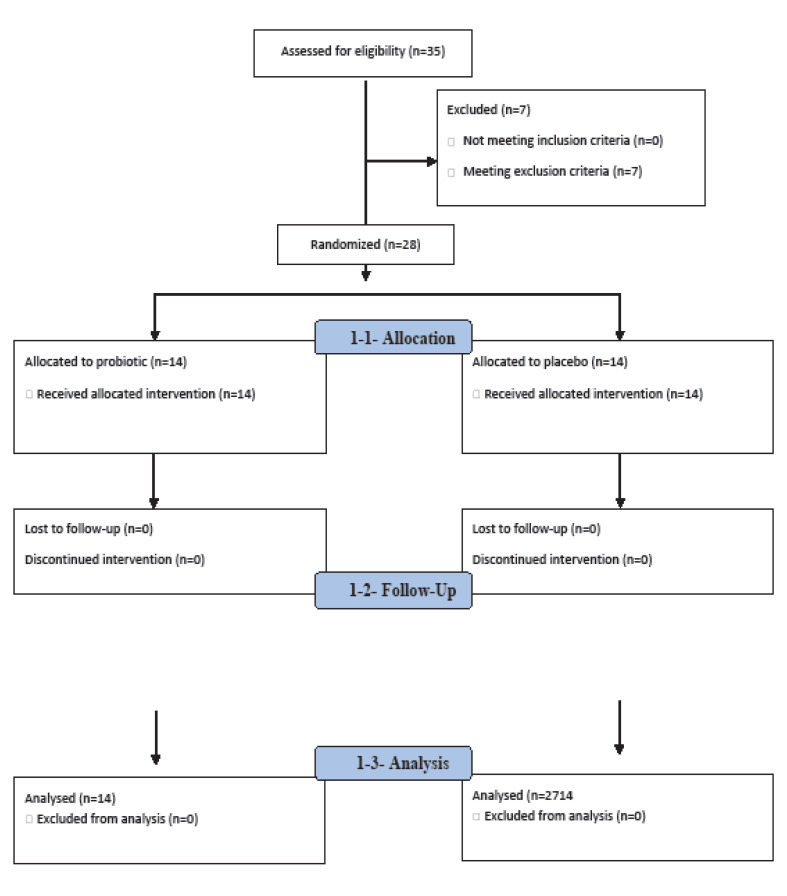
CONSORT flowchart

 The mean age of patients in the probiotics group was 12.08±34.15 years and in the control group was12.32±29.64 years. Figure-1 shows the distribution of gender between two groups (P:0.103). There were no significant differences in occupational exposure to pollutants and smoking in both groups. Distribution of residency in town and rural was the same in the probiotics and control groups without satisfactory differences between two groups. Evaluation of two types of AR in our study showed that 50% (n=7) of patients in the probiotics group and 14.3% (n=2) of patients in the control group had intermittent AR. On the other hand, persistent AR was diagnosed in 86.7 % (n=12) of patients in probiotics group and 50 % (n=7) of patients in the control group, respectively. Past medical history of asthma was observed in 35.7% (n=5) of patients in the probiotics group and 50% (n=7) of patients in the control group. Also, eczema was observed in 50 % (n=7) of patients in the control group and 35.7% (n=5) of patients in the probiotics group and urticaria that was equal in both groups of patients 14.3 % (n=2). According to Table-1, in the probiotics group, 14.3% of patients had sneezing before the start of treatment, but after received probiotic, only 4.6% of patients had sneezing, which shows significant differences in comparison with the control group (P<0.01). The daily dose of nasal corticosteroid consumed in the probiotic group was significantly lower than the control group (P<0.01). Also, the dose of oral corticosteroids in asthmatic patients was significantly reduced in the probiotics group in comparison with the control group (P<0.01). Although cough was present in 7.1% of patients in the probiotics group before starting treatment and then disappeared after probiotics intake, this reduction was not significant between two groups (P=0.31). In the probiotics group, 7.1% of the patients had a night sleep disorder that did not improve after treatment (Figure-2).

## Discussion

AR is a chronic inflammatory disease that affects more than 40% of peoples in the world. Treatments of AR are pharmacological and nonpharmacological. The most common pharmacologic treatments are intranasal corticosteroids, H 1 receptor antagonists (antihistamines), and leukotriene receptor antagonists (6). Low microbial diversity in the gut of pediatrics, especially in children younger than one-year-old is correlated with high occurrence of asthma and allergy and low amounts of *Lachnospira, Veillonella, Faecalibacterium*, and *Rothia* in their gut. Some investigations have shown that probiotic administration can improve the intestinal balance of microorganisms and improving inflammatory conditions [7]. Several studies also have shown that live bacteria in our body are not only harmful but also are useful, and dysfunction of these microbes may correlate with autoimmune diseases (7, 8). Helin *et al*. [9] in a study mentioned that probiotic supplements have no effects on respiratory and ocular symptoms in patients with seasonal allergic rhinitis. In t study by Leu *et al*. probiotics were used as a supplement alongside antihistamines in patients with AR for 24 weeks. After 24 weeks, symptoms of both control and intervention groups were significantly reduced, although in the first 12 weeks the alteration in symptoms in the interventional group was more significant than the control group, in the second 12 weeks the results of examination showed no significant differences between two groups, which the results are similar to the results of our study [10]. According to the findings of our study, there was a significant reduction in the lower conchae hypertrophy, nasal polyp shrinking, and nasal and posterior throat changes after consumption of probiotics, but there were no significant differences between two groups. Coughing, nasal secretion, sleep disturbances, open mouth breathing, night snoring, and posterior throat secretions were not significantly different between the two groups. Xiao *et al*. (9) noted a significant decrease in the nasal secretion of patients that received probiotics, which did not similar to our study. As support to these results, in the study of Nishimura *et al*. (10), nasal symptoms in patients treated with probiotics were significantly less than before treatment. Also, clinical signs and radiographic findings were assessed in a meta-analysis study by Guvenc *et al*. (11). In another study, Jalali *et al*. showed that the addition of probiotics to nasal corticosteroid drugs of persistent AR significantly improved the quality of life of their patients [14]. However, there are some limited studies with definite outcomes in investigating adults with persistent AR. Therefore, our study can be useful for the survey of the therapeutic effects of probiotics in patients with AR and reveals the possibility of using probiotics in adult patients with AR with scrutinies. As observed in the studies, there was a discrepancy between the results of previous studies. An important part of this difference is the kind of probiotics that were used. Because there are many probiotic species with various beneficial effects that lead to different results. On the other hand, the probiotic used in the present study was a commercial probiotic mixed with different bacterial species for the treatment of AR patients. Despite the important findings, this clinical trial study had some limitations. The short duration of intervention was one of the factors that could overcome the effect of achieving adequate therapeutic effects. Also, the small sample size was our limitation in this study.

## Conclusion

 Our results revealed that probiotics have not clear therapeutic effects on AR patients in a short time of treatment. It seems that the duration of therapy and different species of bacteria may influence the efficacy of probiotics in the treatment of AR patients.

## Conflict of Interest

 There is no conflict of interest with the authors.

**Table 1:** Frequency Distribution and Comparison of Treatment Characteristics of Patients

**Variable**	**Probiotics group ** **n(%)**	**Placebo group n(%)**	P-value*
Size decreasing of inferior concha hypertrophy	12(85.7)	13(92.9)	
	2(14)	1(7.1)	0.541
The nasal polyp shrinks	12(85.7)	14(100)	
	2(14)	0(0)	0.481
Nasal discharge changes	12(85.7)	14(100)	
	2(14.3)	0(0)	0.481
CXR changes	13(92.9)	14(100)	0.309

*independent T-test CXR: Chest x-ray

## References

[1] Mårtensson A, Abolhalaj M, Lindstedt M, Mårtensson A, Olofsson TC, Vásquez A (2017). Clinical efficacy of a topical lactic acid bacterial microbiome in chronic rhinosinusitis: a randomized controlled trial. Laryngoscope Investig Otolaryngol.

[2] Lin W-Y, Fu L-S, Lin H-K, Shen C-Y, Chen Y-J (2014). Evaluation of the effect of Lactobacillus paracasei (HF A00232) in children (6–13 years old) with perennial allergic rhinitis: a 12-week, double-blind, randomized, placebo-controlled study. Pediatr Neonatol.

[3] Peng Y, Li A, Yu L, Qin G (2015). The role of probiotics in prevention and treatment for patients with allergic rhinitis: A systematic review. Am J Rhinol Allergy.

[4] Zajac AE, Adams AS, Turner JH, editors. A systematic review and meta-analysis of probiotics for the treatment of allergic rhinitis. International forum of allergy & rhinology; 2015: Wiley Online Library. 10.1002/alr.21492 PMC4725706 25899251

[5] Jan R-H, Chen C-J, Chen L-K, Wen S-H, Lin T-Y (2011). Is the effect of probiotics on allergic rhinitis confined to Dermatophagoides farinae, Dermatophagoides pteronyssinus, or dust-sensitive children? A randomized prospective double-blind controlled trial. Ci Ji Yi Xue Za Zhi.

[6] May JR, Dolen WK (2017). Management of allergic rhinitis: a review for the community pharmacist. Clinical therapeutics.

[7] Anand S, Mande SS (2018). Diet, microbiota and gut-lung connection. Front Microbiol.

[8] Ipci K, Altıntoprak N, Muluk NB, Senturk M, Cingi C (2017). The possible mechanisms of the human microbiome in allergic diseases. Eur Arch Otorhinolaryngol.

[9] Xiao JZ, Kondo S, Yanagisawa N, Takahashi N, Odamaki T, Iwabuchi N (2006). Probiotics in the treatment of Japanese cedar pollinosis: a double-blind placebo-controlled trial. Clin Exp Allergy.

[10] Nishimura I, Igarashi T, Enomoto T, Dake Y, Okuno Y, Obata A (2009). Clinical efficacy of halophilic lactic acid bacterium Tetragenococcus halophilus Th221 from soy sauce moromi for perennial allergic rhinitis. Allergol Int.

[11] Güvenç IA, Muluk NB, Mutlu FŞ, Eşki E, Altıntoprak N, Oktemer T (2016). Do Probiotics have a role in the Treatment of Allergic Rhinitis? A Comprehensive Systematic Review and Metaanalysis. Am J Rhinol Allergy.

